# Compressive Strength Estimation of Steel-Fiber-Reinforced Concrete and Raw Material Interactions Using Advanced Algorithms

**DOI:** 10.3390/polym14153065

**Published:** 2022-07-29

**Authors:** Kaffayatullah Khan, Waqas Ahmad, Muhammad Nasir Amin, Ayaz Ahmad, Sohaib Nazar, Anas Abdulalim Alabdullah

**Affiliations:** 1 Department of Civil and Environmental Engineering, College of Engineering, King Faisal University, Al-Ahsa 31982, Saudi Arabia; mgadir@kfu.edu.sa (M.N.A.); 218038024@student.kfu.edu.sa (A.A.A.); 2 Department of Civil Engineering, COMSATS University Islamabad, Abbottabad 22060, Pakistan; waqasahmad@cuiatd.edu.pk (W.A.); sohaibnazar@cuiatd.edu.pk (S.N.); 3 MaREI Centre, Ryan Institute and School of Engineering, College of Science and Engineering, National University of Ireland Galway, H91 HX31 Galway, Ireland; a.ahmad8@nuigalway.ie

**Keywords:** SFRC, building material, compressive strength, steel fiber, concrete

## Abstract

Steel-fiber-reinforced concrete (SFRC) has been introduced as an effective alternative to conventional concrete in the construction sector. The incorporation of steel fibers into concrete provides a bridging mechanism to arrest cracks, improve the post-cracking behavior of concrete, and transfer stresses in concrete. Artificial intelligence (AI) approaches are in use nowadays to predict concrete properties to conserve time and money in the construction industry. Accordingly, this study aims to apply advanced and sophisticated machine-learning (ML) algorithms to predict SFRC compressive strength. In the current work, the applied ML approaches were gradient boosting, random forest, and XGBoost. The considered input variables were cement, fine aggregates (sand), coarse aggregates, water, silica fume, super-plasticizer, fly ash, steel fiber, fiber diameter, and fiber length. Previous studies have not addressed the effects of raw materials on compressive strength in considerable detail, leaving a research gap. The integration of a SHAP analysis with ML algorithms was also performed in this paper, addressing a current research need. A SHAP analysis is intended to provide an in-depth understanding of the SFRC mix design in terms of its strength factors via complicated, nonlinear behavior and the description of input factor contributions by assigning a weighing factor to each input component. The performances of all the algorithms were evaluated by applying statistical checks such as the determination coefficient (R^2^), the root mean square error (RMSE), and the mean absolute error (MAE). The random forest ML approach had a higher, i.e., 0.96, R^2^ value with fewer errors, producing higher precision than other models with lesser R^2^ values. The SFRC compressive strength could be anticipated by applying the random forest ML approach. Further, it was revealed from the SHapley Additive exPlanations (SHAP) analysis that cement content had the highest positive influence on the compressive strength of SFRC. In this way, the current study is beneficial for researchers to effectively and quickly evaluate SFRC compressive strength.

## 1. Introduction

The mechanical properties, toughness, ductility, fatigue resistance, and crack-arresting of concrete can be improved by adding fibers into it [1,2,3,4,5,6,7,8,9]. Specifically, the addition of steel fibers to cementitious concrete enhances the post-cracking behavior and toughness [10,11,12,13,14,15]. The addition of adequate steel fiber content (i.e., 0–1.5%) to concrete can improve its properties [1]. Steel, artificial, and natural fibers are incorporated into concrete to enhance the mechanical properties and resistance against cracks of cementitious concrete composites [2,3,4,5,6,7,8]. Different studies have been conducted on models for regular concrete mechanical properties depending on a wide database [16], although there are additional predicting parameters such as fiber type, aspect ratio, and volumetric content for SFRC compared to normal concrete. However, the development of appropriate predictive models is still new. Subsequently, the scuffling of conventional nonlinear and linear regression models is used to determine the compressive strength of SFRC. ML techniques may assist in resolving the issue of difficulty for the strength prediction of SFRC [9,10,11,12,13,14,15,16]. Although multiple experimental studies have been conducted for this purpose, as reported in the literature, the prediction of SFRC properties having different mix design components is still quite hard. Therefore, in the current study, an effort is made to predict SFRC compressive properties by employing ML approaches.

The employment of ML techniques may effectively resolve complex problems in various engineering fields. Based on an input database, ML techniques may estimate outcomes. To predict concrete properties, two ML approaches, a standalone procedure (a single-model-based decision tree) and ensemble techniques (i.e., random forest, gradient boosting, and XGBoost) are used. As reported in the literature, the performance of ensemble models has been better than individual ML models. Chaabene, et al. [17] evaluated in detail the employment of ML techniques for the prediction of concrete mechanical properties. Furthermore, several types of research have been conducted for the anticipation of mechanical properties of different concrete types, such as phase-change-material-integrated concrete [18], self-healing concrete [19], high-performance concrete (HPC) [20], recycled aggregate concrete (RAC) [21], etc. The employment of ML techniques was performed by Han, et al. [22] to predict HPC compressive strength. Fine aggregates, coarse aggregates, cement, water, GGBFS, age, and five additional variable combinations were all considered as the database for input factors. The developed model provided HPC compressive strength prediction with high precision.

SFRC mechanical properties have extensively been determined in various studies [23,24,25]. However, the procedures of specimen-casting in the laboratory, curing, and testing consume a lot of time, effort, cost, and labor. Therefore, the employment of ML modern techniques for the assessment of SFRC mechanical properties may resolve such issues and reduce expenses for experimentation. Moreover, the effects of raw ingredients on compressive strength have still not been addressed considerably in recent research, providing a research gap. Accordingly, the effects of raw materials, i.e., input factors, on the outcome factor, i.e., compressive strength, are also determined and explained by performing SHapley Additive exPlanations. In the current study, different ensemble ML techniques are applied to predict SFRC compressive strength. Random forest, gradient boosting, and XGBoost are employed as ensemble ML models. Furthermore, a statistical analysis is also performed to evaluate all the models, and different ML models are compared. Based on the performances, a superior model is proposed to predict SFRC compressive strength. In addition, a SHapley Additive exPlanations (SHAP) analysis, i.e., a post hoc model agnostic approach, is employed to gain insight into the ML models [26,27]. SHAP integration with the ML algorithms is also performed in this study, which is still a research gap. The purpose of a SHAP analysis is to give an in-depth understanding of the SFRC mix design in terms of its strength factors via complex, nonlinear behavior and the description of input factor contributions by allocating a weighing factor to every input factor. It assists in the development of sustainable and durable concrete mixes.

## 2. Methodology

### 2.1. Machine-Learning Techniques

**Figure 1:**
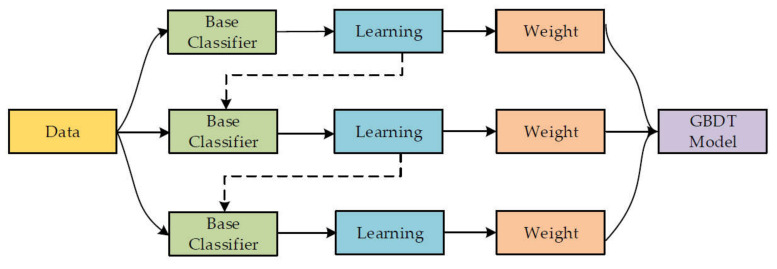
Gradient-boosting training process [29].

This ensemble method for classification and regression was proposed by Friedman [28]. The gradient-boosting method is the same as other boosting techniques but is limited to regression only. In this technique, each training set iteration is selected randomly and is validated by the base model, as represented in Figure 1. The execution accuracy and speed of gradient boosting can be enhanced by randomly subsampling the training data, which ultimately helps to avoid overfitting. The smaller the training data fraction, the higher the regression speed to fit smaller model data at each iteration. A shrinkage rate and an n-tree tuning factor are needed in gradient-boosting regression, where n-tree denotes the number of grown trees. Here, the n-tree value should not be too small, and the shrinkage factor, usually named as the learning rate, is applicable for each expansion tree.

Chen and Guestrin [30] proposed an extreme gradient-boosting (extreme gradient boosting) algorithm, which is considered an authentic tool for researchers in the data science field due to the effective tree-based ensemble learning algorithm. Gradient-boosting architecture, i.e., applying different functions for result estimation using Equation (1), is the basis of extreme gradient boosting [28]:

(1) $${\bar{y}}_{i}=y_{i}^{0}+ɳ\sum_{K=1}^{n}f_{k}\left(U_{i}\right)$$

where the predicted output is shown by ${\bar{y}}_{i}$, using ith data with $U_{i}$ as a parameter vector; n shows the estimator quantity in correspondence with independent tree structures against every $f_{k}$, where the range of k is from 1 to n; and $y_{i}^{0}$ is the main hypothesis (mean of the original factors in the training dataset). η depicts the learning rate to enhance the model performance, along with the connection of additional trees to avoid overfitting. One major conflict in ML is developing a model with the least amount of overfitting. The training phase is complementarily evaluated in the extreme gradient-boosting model.

**Figure 2:**
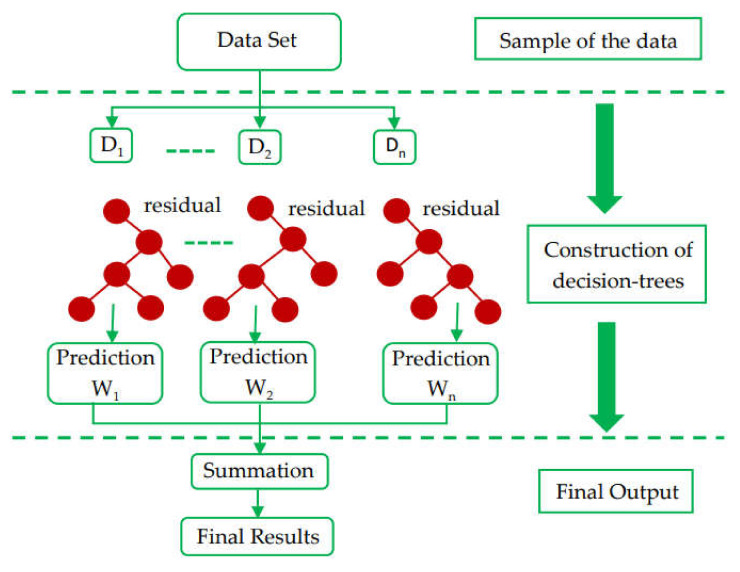
XGBoost (extreme gradient-boosting) algorithm structure [31].

As per Equation (1), at the k^th^ level, the k^th^ estimator is in connection with the model, the forecasting of the k^th^ $y_{i}^{-k}$ is determined through the predicted output $y_{i}^{-\left(k-1\right)}$ in a further step, and the respective developed f_k_ against the k^th^ complementary estimator is provided in Equation (2):

(2) $$y_{i}^{-k}=y_{i}^{-\left(k-1\right)}+ɳf_{k}$$

where f_k_ depicts the weight of the leaves and is developed by minimizing the k^th^ tree objective function (Equation (3)):

(3) $$f_{\mathrm{obj}}=\gamma Z+\sum_{a=1}^{Z}\left[g_{a}\omega_{a}+\frac{1}{2}\left(h_{a}+\lambda \right)\omega_{a}^{2}\right]$$

where the leaf node quantum is denoted by Z, the complexity factor by c, the constant coefficient by λ, and the weight (i.e., 1−Z) of the leaf by $\omega_{a}^{2}$. λ and c are controlling factors applied to improve the model in terms of avoiding overfitting. $h_{a}$ and $g_{a}$ are the summed factors for the whole dataset linked with the previous and initial loss function gradient leaves, respectively. For building the k^th^ tree, a leaf is further bifurcated into multiple leaves. Gain parameters are used to apply such a system, as given in Equation (4):

(4) $$G=\frac{1}{2}\left[\frac{O_{L}^{2}}{P_{L}+\lambda }+\frac{O_{R}^{2}}{P_{R}+\lambda }+\frac{{\left(O_{L}+O_{R}\right)}^{2}}{P_{L}+P_{R}+\lambda }\right]$$

where the gain parameters are denoted by G, and the right and left leaves are PR and OR, as well as P_L_ and O_L_, respectively. The division criteria are generally assumed when approximating the gain parameter at zero. λ and c are controlling factors that are dependent indirectly on the gain parameters. For instance, the gain parameter can considerably be decreased by a larger regularization parameter, ultimately preventing the leaf convolution process. However, the model performance for adopting training data is also be reduced by this. The basic, level-wise structure of the extreme gradient boosting tree model is shown in Figure 2.

**Figure 3:**
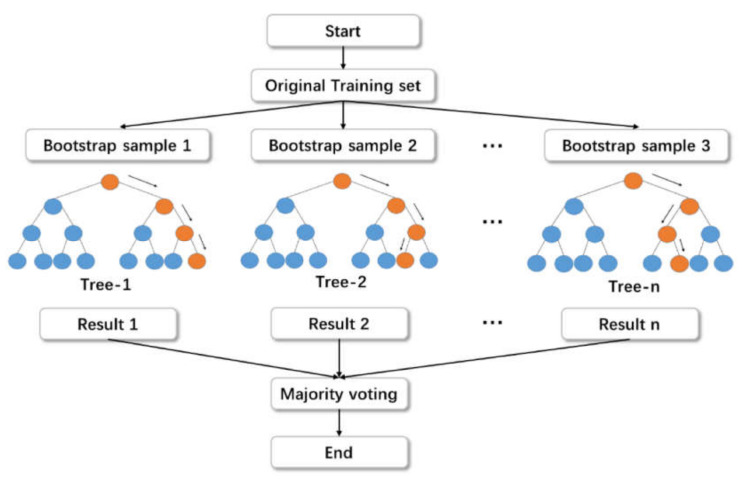
Random forest algorithm structure [35].

The random forest model is a regression- and classification-based approach that has been studied by various researchers [22,32]. The compressive strength of concrete is predicted using a random forest model, as performed by Shaqadan [33]. The prime difference between random forest and DT is the number of trees. A single tree is developed in DT; however, in random forest, multiple trees are built, which is known as a forest. The dissimilar data are selected arbitrarily and are accordingly allocated to respective trees. Each tree has data in rows and columns, and different dimensions for the rows and columns are determined. The following steps are carried out for the growth of each tree: The data frame comprises 2/3 of the whole dataset that is randomly selected for each tree. This method is known as random forest. Random selection is made for the prediction variables, and the node splitting is achieved by finely splitting these variables. For all the trees, the remaining data are utilized to estimate the out-of-bag error. Accordingly, the final out-of-bag error rate is assessed by combining errors from each tree. Each tree provides regression, and among all the forest trees, the forest with the greatest amount of votes is selected for the model. The value of the votes can either be 1 or 0. The obtained proportion of 1 specifies the prediction probability. Among all the ensemble algorithms, random forest is the most sophisticated one. It includes desirable features for variable importance measures (VIMs) with robust overfitting resistance and fewer model parameters. DT is used as a base predictor for random forest. Acceptable results can be produced by random forest models with default parameter settings [34]. As allowed by random forest, combinations of parameter settings and base predictors can be reduced to one. The basic, level-wise structure of the random forest model is presented in Figure 3.

Furthermore, the current study identified influences for a global feature, as well as the interactions and dependencies of the considered feature on SFRC, depending on a game theory technique named SHapley Additive exPlanations (SHAP) [36] to enhance the explainability of the proposed model. In this technique, the prediction of every instance was explained by the computation of contributions for all the considered features for forecasting by applying Shapley values from the game theory coalition. The contribution for each individual feature value over all the possible combinations was marginally averaged to produce the Shapley value. The more influential features had higher absolute Shapley values. The Shapley values against each feature from the database were averaged to attain global feature influences. Afterwards, the sorting of these values in a decreasing manner in terms of importance was performed, followed by their plotting. A single point on the plot represented a Shapley value against individual instances and features. Feature importance and Shapley values determined the y and x axis positions, respectively. The higher influence of a feature on SFRC was depicted from its higher position on the y-axis, and its importance was depicted from a low-to-high color scale. The interactions of features and the corresponding impacts on SFRC were depicted from the SHAP feature dependence plots, in which interactions with other features were colored. This process provides better information than conventional plots of partial dependence [37]. In SHAP, more specifically, the feature importance (*j*) for the output of the model f $\phi^{j}\left(f\right)$, is allocated weightage for the summation of feature contributions towards the model outcome $f\left(x_{i}\right)$ to gain the overall possible feature combinations [38]. The $\phi^{j}\left(f\right)$ is expressed by Equation (5), as provided below:

(5) $$\phi^{j}\left(f\right)=\sum_{S\subseteq \left\{x^{1},\ldots ..,x^{p}\right\}/\left\{x^{j}\right\}}\frac{\left|S\right|!\left(p-\left|S\right|-1\right)!}{p!}\left(f\left(S⊔\left\{x^{j}\right\}\right)-f\left(S\right)\right)$$

where

*S* is the feature subset;
$x_{j}$ is the feature *j*;
*p* is the feature number in the model.

In the SHAP technique, the feature importance is determined by quantifying prediction errors while disturbing a specific feature value. The sensitivity of the prediction error is taken to allocate weightage to the significance of the feature while perturbing its value. The performance of the trained machine-learning model was also explained with the help of SHAP. SHAP employs an additional feature attribution technique, i.e., the addition of linear input factors, to demonstrate an interpretable model, which is taken by the model’s outcome. For instance, a model with input parameters $x_{i}$ (in which i ranges between 1 and k, and k depicts the input parameter number) and h ($x_{s}$) depicts the explanation model with $x_{s}$ as a simple input. However, Equation (6) is deployed to portray the original model $f\left(x\right)$:

(6) $$f\left(x\right)=h\left(x_{s}\right)=\emptyset_{0}+\sum_{i=1}^{p}\emptyset_{i}x_{s}^{i}$$

where

p is the input feature number;
$\emptyset_{0}$ is the constant without any information (i.e., no input).

**Figure 4:**
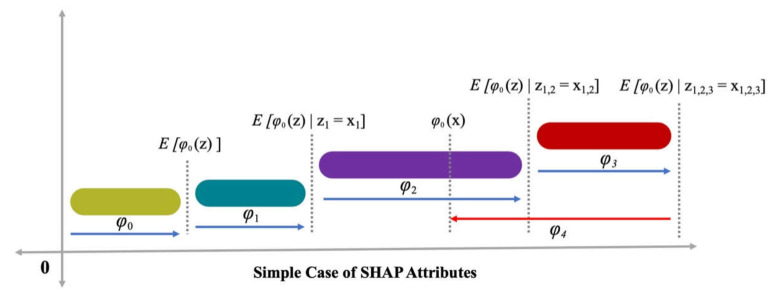
SHAP attributes [40].

$x=m_{x}\left(x_{s}\right)$ indicates that the mapping function has a relationship with both the x and $x_{s}$ input parameters. Lundberg and Lee [39] presented Equation (6) in which (h ()), i.e., the prediction value, was enhanced by the $\emptyset_{0},\;\emptyset_{1},\;and\;\emptyset_{3}$ terms, and a decrease of $\emptyset_{4}$ in the h () value was also observed (Figure 4). A single-value key to Equation (6) is the inclusion of three desired characteristics, such as consistency, missingness, and local accuracy. Consistency ensures no reduction in the attribution and is assigned to the respective feature as a change in a feature of more impact. Missingness ensures no value for importance is assigned to the missing features, i.e., $\emptyset_{i}=0$ is employed by $x_{s}^{i}=0$. Local accuracy ensured that the summation of feature attribution is taken as a function for the outcome, which includes a requirement of the model for matching the outcome f with $x_{s}$ as a simplified input. $x=m_{x}x_{s}$ represents the attainment of local accuracy.

### 2.2. Dataset Description

**Figure 5:**
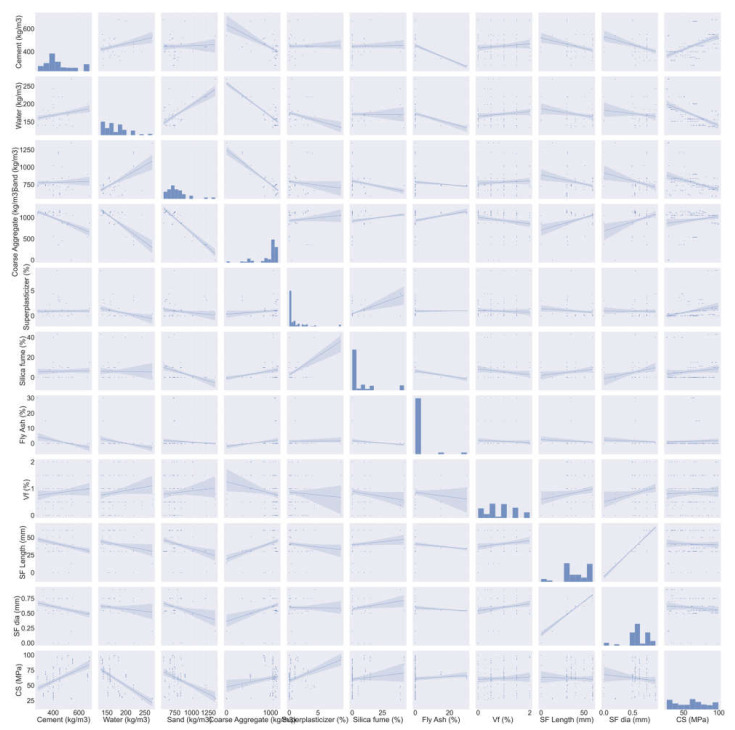
Description of parameter data.

**Figure 6:**
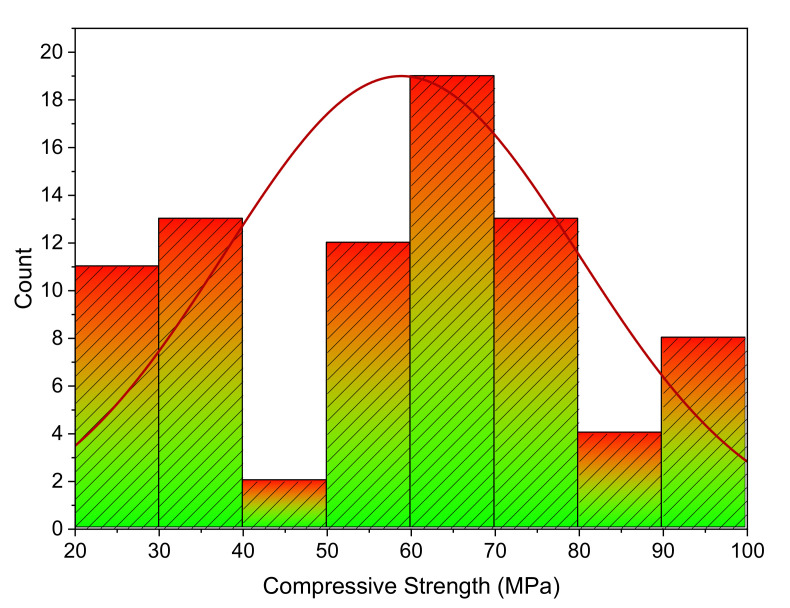
SFRC compressive strength distribution.

The database that was employed for the prediction of SFRC compressive strength is shown in Figure 5. Data regarding the compressive strength of SFRC were extracted from the literature [41,42,43,44,45,46,47,48,49,50,51,52,53,54,55,56,57]. These included cement, water, sand, coarse aggregates, super-plasticizer, silica fume, fly ash, steel fiber, fiber length, and fiber diameter as inputs. These input factors were considered as compressive strength predictor variables. All these input and output parameters were collected within a compressive strength range of 20–100 MPa. These studies were selected because of the similarities between their input parameters. Figure 5 depicts the range for every variable and the minimum and maximum values. The compressive strength of SFRC was estimated using the Python and Spyder scripting of Anaconda software. The compressive strength histogram taken in the current study is presented in Figure 6.

## 3. Results and Discussion

### 3.1. XGBoost

**Figure 7:**
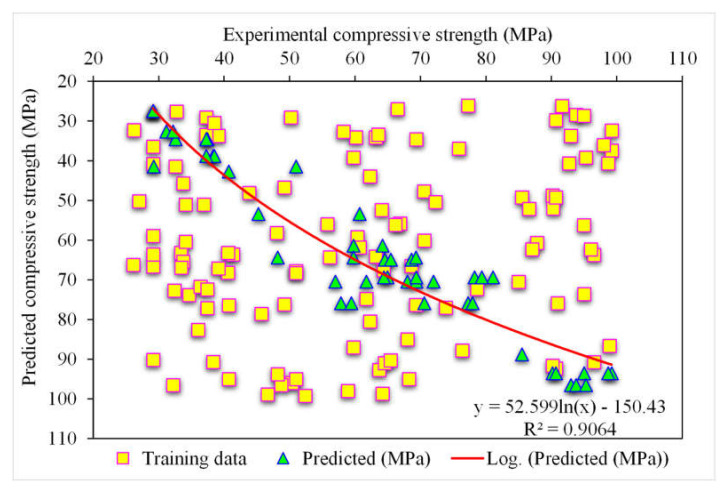
Experimental and XGBoost predicted results.

**Figure 8:**
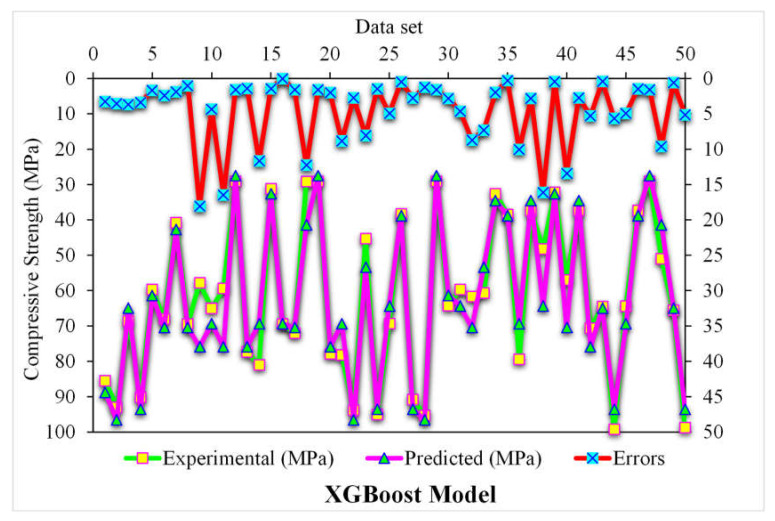
Experimental and XGBoost predicted values with errors.

Figure 7 depicts the predicted and experimental value comparison for SFRC compressive strength using the XGBoost algorithm. A highly accurate outcome prediction for SFRC compressive strength was provided by the XGBoost algorithm. The adequacy of the XGBoost model was specified with the satisfactory R^2^ value of 0.90. The error distribution of XGBoost was predicted, and experimental values for SFRC compressive strength are illustrated in Figure 8. The average error value for SFRC compressive strength was 4.63 MPa. A total of 70% of the total error values were below 5 MPa; 16% of these values ranged between 5 and 10 MPa, and 14% were above 10 MPa.

### 3.2. Gradient Boosting

**Figure 9:**
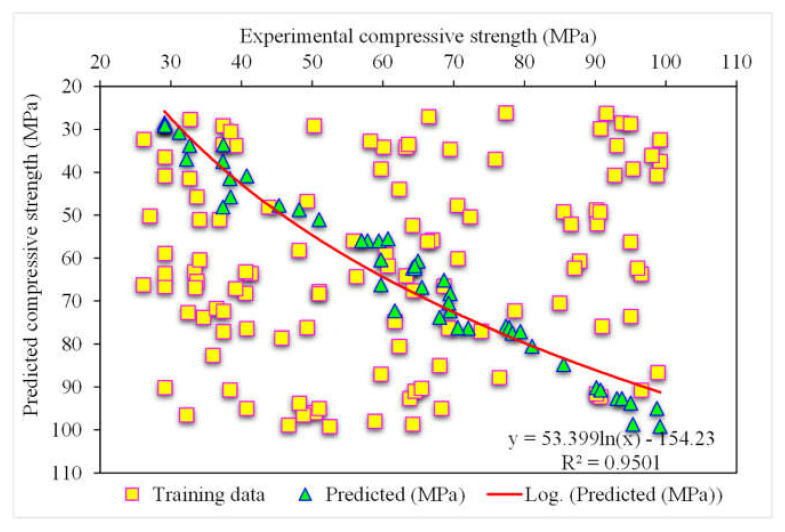
Experimental and gradient boosting predicted results.

**Figure 10:**
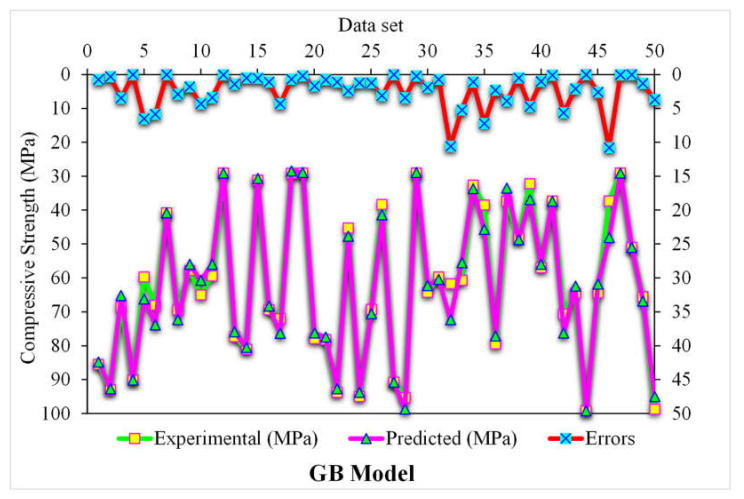
Experimental and gradient boosting predicted values with errors.

Figure 9 depicts the estimated gradient-boosting model and experimental outcome values for SFRC compressive strength. The 0.95 R^2^ value in the case of gradient boosting showed outcomes with higher accuracies than the XGBoost model. Figure 10 shows the error distribution for the gradient boosting estimated and experimental values in the case of SFRC compressive strength. It can be observed that 86% of the values were less than 5 MPa, 10% were between 5 and 10 MPa, and the remaining 4% of the values were above 10 MPa. The higher R^2^ and lesser error values represented the higher precision of gradient boosting than the XGBoost model.

### 3.3. Random Forest

**Figure 11:**
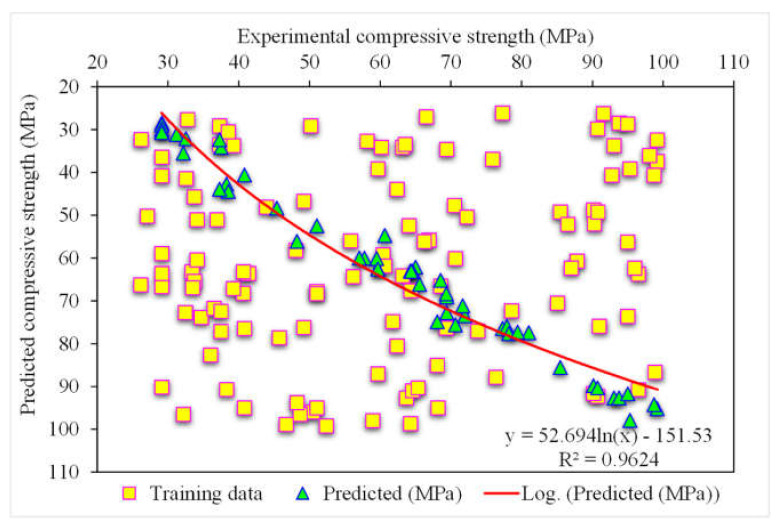
Experimental and random forest predicted results.

**Figure 12:**
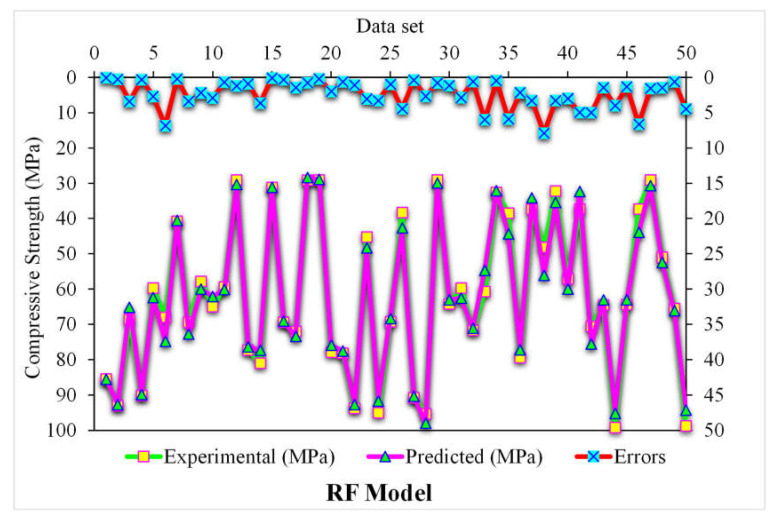
Distribution of experimental and BSA predicted values with errors.

Figure 11 demonstrates the random forest estimated outcomes and experimental values for SFRC compressive strength. The 0.96 R^2^ value for the random forest model represented more adequate results than the other two models. At the same time, the estimated SFRC compressive strength outcomes in the case of random forest were preferable to all the other considered ensemble models. Figure 12 reveals the distribution of random forest predicted and experimental values with errors for SFRC compressive strength. Here, 90% of the error values were less than 5 MPa, and the remaining 10% of the values were between 5 and 10 MPa. At the same time, not a single error value was more than 10 MPa. The R^2^ and error values for SFRC compressive strength in the case of random forest were more precise and acceptable. Therefore, this outcome indicated that high-precision results could be predicted using random forest compared to other models.

### 3.4. Comparison of All Models

**Figure 13:**
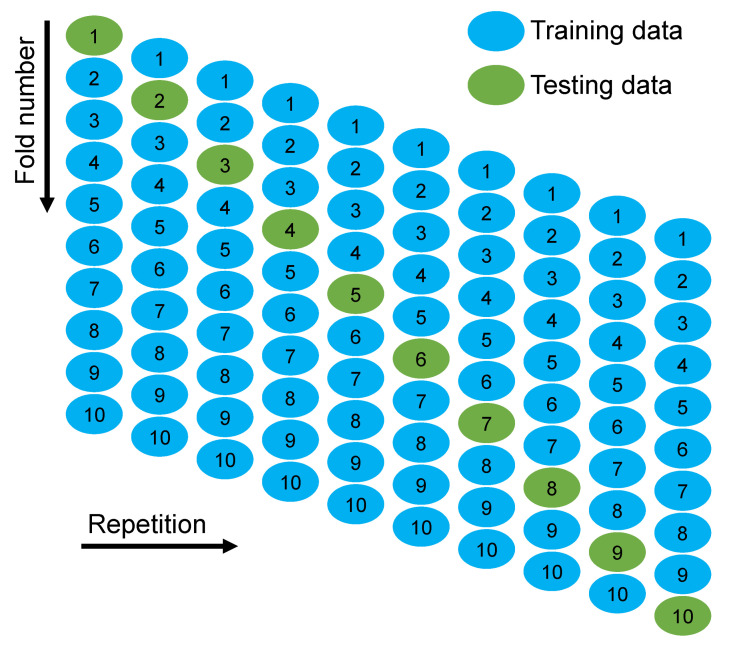
K-fold cross-validation procedure [62].

**Figure 14:**
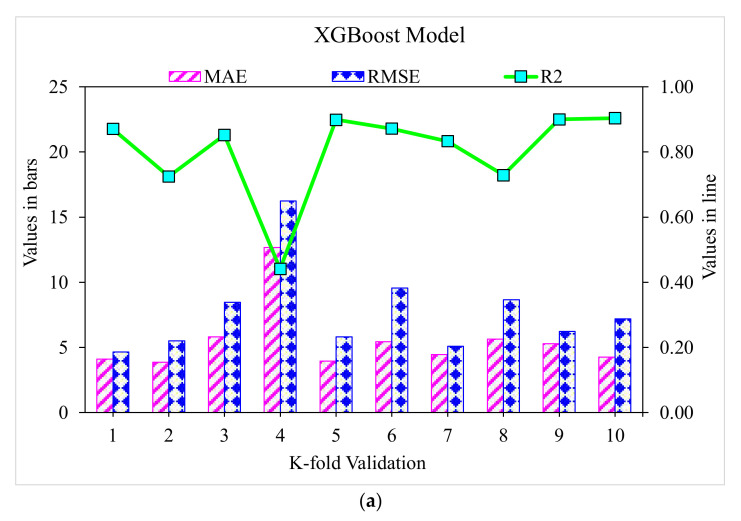

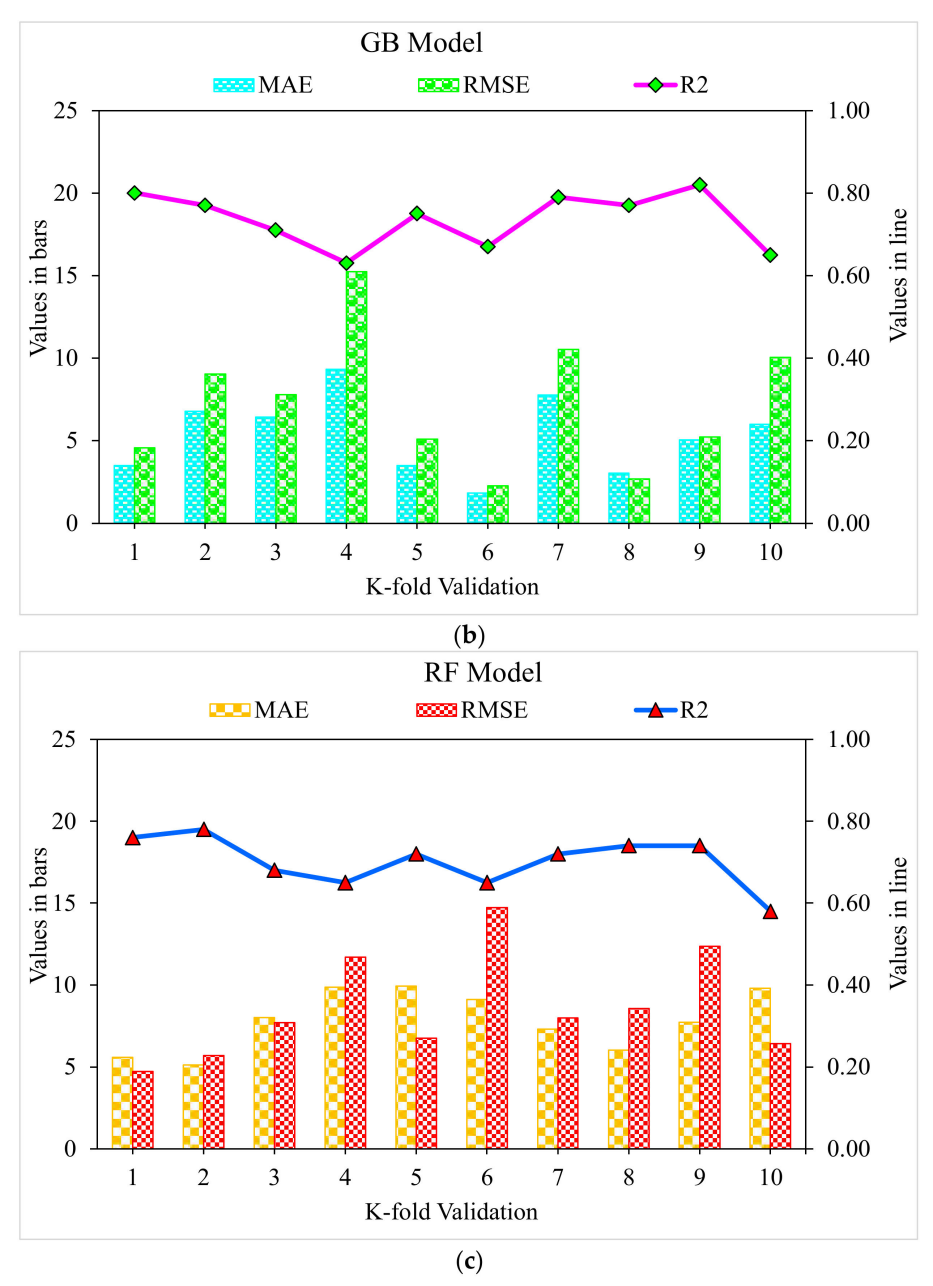
Statistical representation: (**a**) XGBoost; (**b**) gradient boosting; and (**c**) random forest.

A k-fold cross-validation technique was adapted to validate the executing model. The model’s performance was assessed by employing statistical checks [58,59,60,61]. Generally, the data were split into ten groups with random dispersion to perform k-fold cross-validation, and the repetition of this method was made ten times to attain results within an acceptable range, as presented in Figure 13. The statistical checks are listed in Table 1 for all the models. The R^2^ values for the random forest, gradient-boosting, and XGBoost models were 0.96, 0.95, and 0.90, respectively, as illustrated in Figure 14a–c. The R^2^ value for random forest was more than the other considered models, having less error values for SFRC compressive strength.

**Figure 15:**
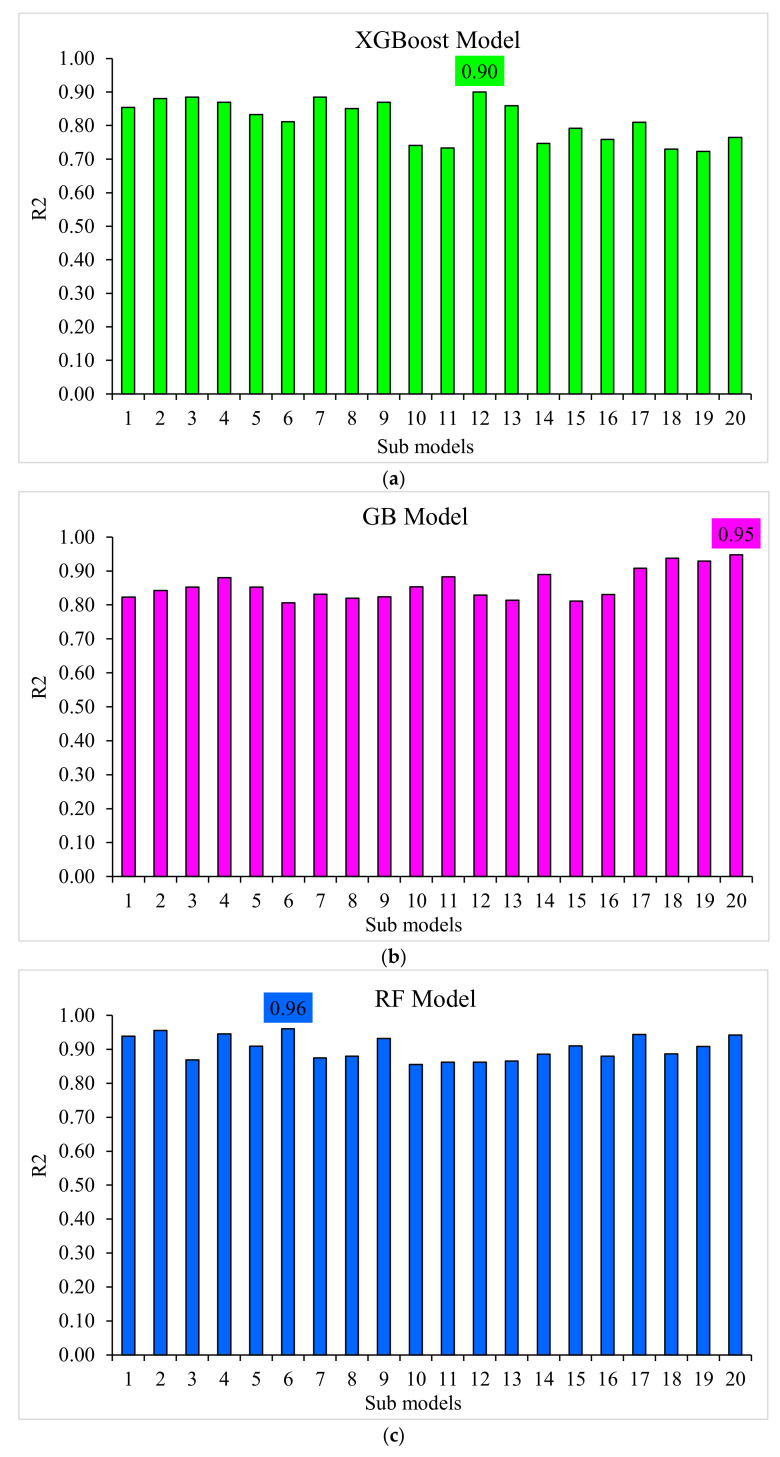
Submodel results: (**a**) XGBoost; (**b**) gradient boosting; and (**c**) random forest.

The SFRC compressive strength was estimated by employing the ensemble ML techniques in the current study to offer reliable and efficient results. The 0.96 R^2^ value in the case of the random forest outcome showed a precise estimation of SFRC compressive strength. The superiority of the ensemble random forest ML algorithms for the compressive strength prediction of SFRC utilizing a single, optimized model out of twenty submodels is depicted in Figure 15a–c. Hence, it can be summarized that random forest showed higher precision and lower errors than the other considered models.

### 3.5. Enhanced Explainability of ML Models

**Figure 16:**
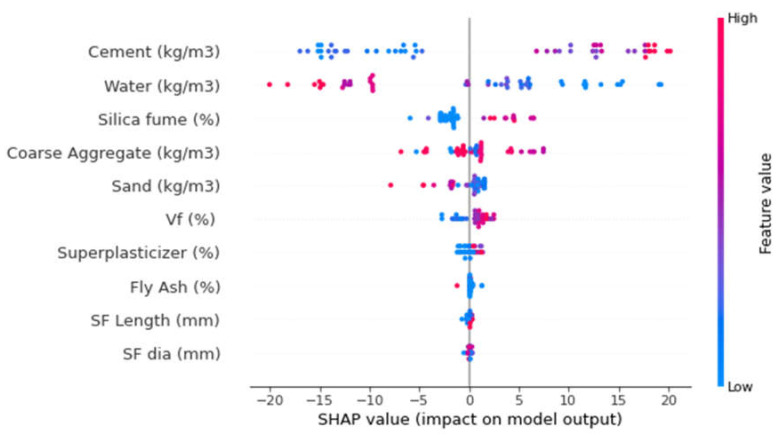
SHAP plot.

**Figure 17:**
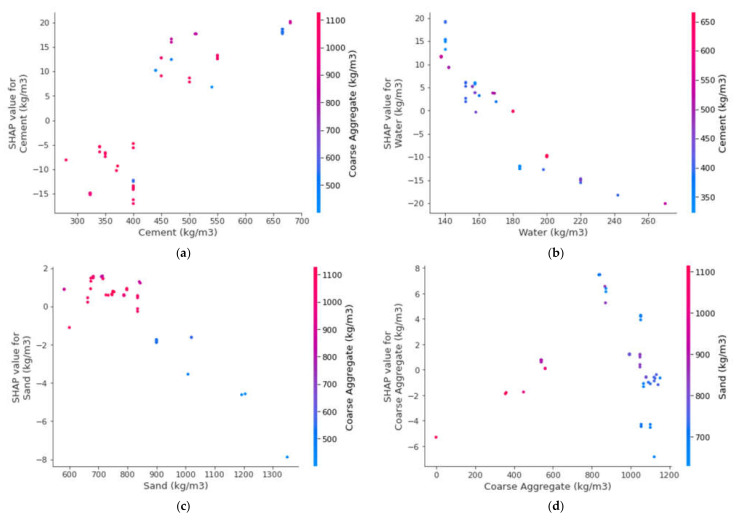

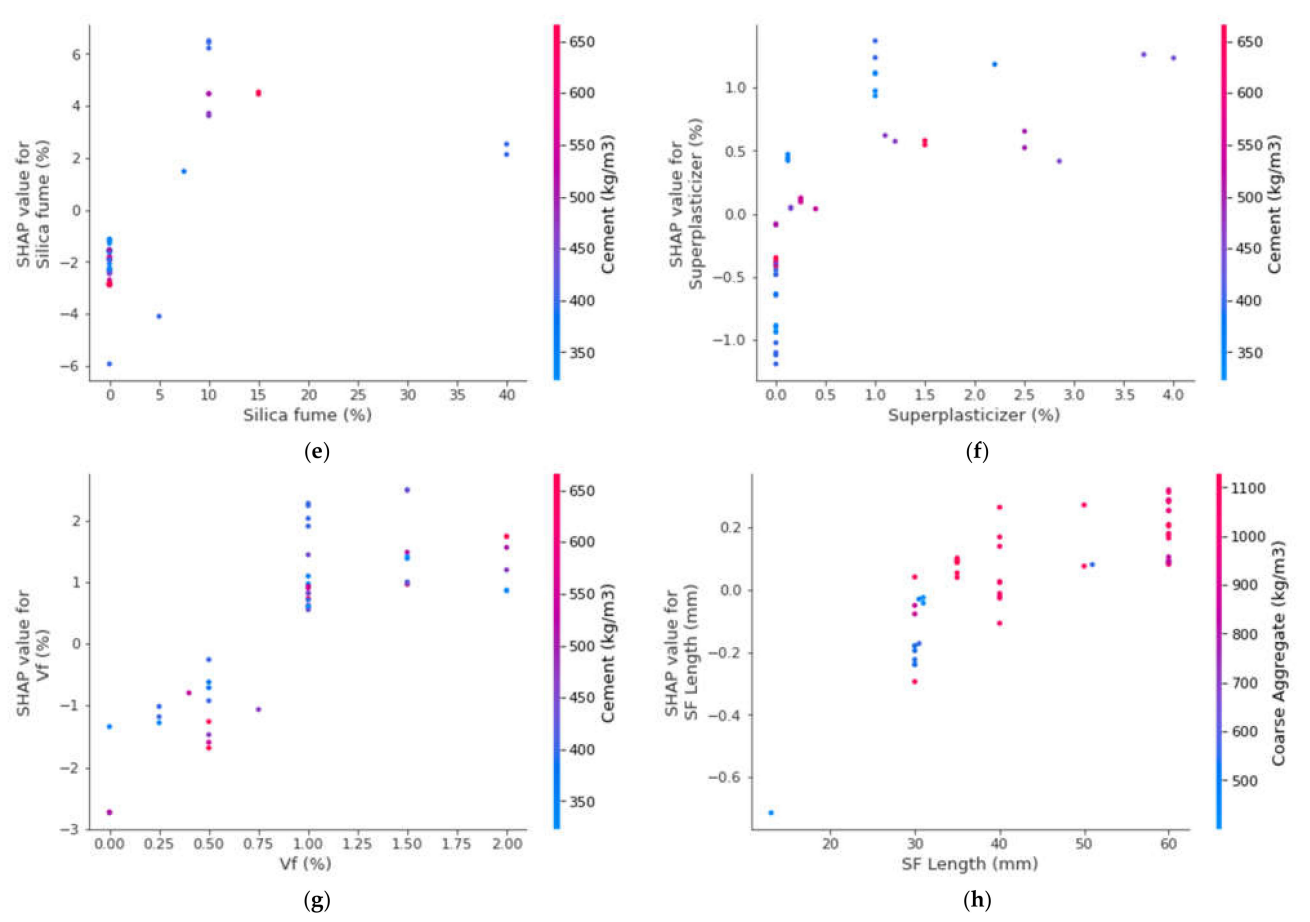
Interaction plots of various parameters: (**a**) cement; (**b**) water; (**c**) sand; (**d**) coarse aggregates; (**e**) silica fume; (**f**) super-plasticizer; (**g**) V_f_; and (**h**) fiber length.

This study also presents a detailed explanation of machine-learning models, as well as the interactions and dependencies of all the input features. By employing a SHAP tree explainer for the whole database, an enhanced feature that influences global representation is presented by merging local SHAP explanations. TreeExplainer, i.e., a tree-like SHAP approximation approach, was applied [63]. In this process, tree-based models’ internal structures were assessed, which is the summation of the calculations linked with a tree model leaf node, leading to low-order complexity [63]. As the XGBoost model provided highly precise SFRC compressive strength prediction, this section interprets the model for SFRC compressive strength with the help of SHAP analysis. The different features were correlated with SHAP values for the SFRC compressive strength (as acquired from ensemble XGBoost modeling), as illustrated in Figure 16. It may be noted that the cement feature had the highest, i.e., approximately 20, SHAP value for SFRC compressive strength prediction. The cement feature positively influenced the SFRC compressive strength, which means that, by increasing the cement content, its strength was enhanced. The second-highest SHAP value was for water against SFRC compressive strength; however, it negatively influenced it. Enhancing the water content resulted in reduced SFRC compressive strength and vice versa. Afterwards, silica fume, the main factor for SFRC, had a SHAP value of approximately 5 (Figure 17). The silica fume content positively influenced the compressive strength of SFRC. Increasing the content of silica fume turned into increased SFRC compressive strength. Then, coarse aggregates were the next in terms of SHAP value. However, in this scenario, enhancement in the SFRC compressive strength resulted in the optimum content of coarse aggregates. After the optimized content, with any further addition of coarse aggregate content, the compressive strength of the SFRC decreased. In both ways, i.e., positive and negative, the influence of coarse aggregates on the compressive strength of SFRC was demonstrated by this behavior, whereas, in the case of fine aggregates, a negative influence on the compressive strength of SFRC was observed. SFRC particle packing density was difficult to attain in the case of an enhanced content of sand. In the same way, the SHAP value for fiber volume was next, followed by super-plasticizer, and silica fume, as well as steel fiber length and diameter. More or less the same SHAP values near zero were observed for all these features, depicting their lesser influence on SFRC compressive strength.

The different features’ interactions with SFRC compressive strength are illustrated in Figure 17. The interaction of the cement feature is presented in Figure 17a. It may be noted from the plot that cement influenced the SFRC compressive strength and was in a direct relationship with it. In Figure 17b, the negative influence of water content on SFRC compressive strength can be observed. The inverse relation of water content with SFRC compressive strength was observed. The interaction of the sand feature is presented in Figure 17c. Due to its effect on silica fume, the sand content depicted a negative influence and resulted in reduced SFRC compressive strength. Then, the coarse aggregates feature depicted both positive and negative interactions, depending upon the content (Figure 17d). The range up to the optimum content resulted in positive interactions, while interactions above that were negative. The silica fume interaction plot is shown in Figure 17e. It depicted an increasing trend of up to 20% content. However, it indicated negative influence at a considerably high content, i.e., 40%. Similarly, super-plasticizer showed a positive influence up to 2% content; however, further content negatively influenced the SFRC compressive strength. Likewise, the steel fiber volumetric content interacted positively and influenced SFRC compressive strength (Figure 17g). Figure 17h shows the interaction of steel fiber length with coarse aggregate content. It depicts a positive influence on SFRC compressive strength with enhancement in the steel fiber length.

## 4. Conclusions

The employment of machine-learning (ML) approaches to predict the mechanical properties of concrete in the construction industry is gaining attention nowadays. The main aim of the current study was to assess the precision of ML techniques to predict the compressive strength of steel-fiber-reinforced concrete (SFRC). The ten input parameters for prediction were cement, fine aggregates (sand), coarse aggregates, water, super-plasticizer, fly ash, silica fume, steel fiber length, and fiber diameter. The following outcomes were drawn from the conducted study:

- The 0.96 R^2^ value in the case of the random forest model showed its accuracy in predicting SFRC compressive strength. In the case of ensemble gradient-boosting and XGBoost ML models having 0.95 and 0.90 R^2^ values, respectively, the predicted SFRC compressive strength had less accuracy.
- The predicted SFRC compressive strength was optimized using twenty submodels with a range of 10 to 200 predictors. The ensemble random forest model produced a comparatively more precise prediction of SFRC compressive strength than all the other considered models.
- As revealed from the k-fold cross-validation outcomes, the gradient-boosting and random forest models had higher R^2^ and lesser RMSE and MAE values for SFRC compressive strength than the other considered models, where the random forest model displayed the best accuracy for SFRC compressive strength prediction.
- Statistical checks such as RMSE and MAE were employed to evaluate the performances of the models. However, the higher determination coefficient and lower error value showed the superiority of the random forest model in the prediction of SFRC compressive strength.
- Among all the ML techniques, the random forest was the best approach to estimate SFRC compressive strength.
- The cement feature had the highest influence on the prediction of SFRC compressive strength, followed by water content, silica fume, coarse aggregates, sand, volumetric fiber content, and content of super-plasticizer, as revealed from SHAP analysis. However, the SFRC compressive strength was least influenced by the diameter of the steel fibers.
- SFRC compressive strength was positively influenced by cement content, as well as steel fiber volumetric content and length, as depicted from the feature interaction plots.

Indeed, a proper relational database and testing are important for engineering applications. This study was limited to the prediction of compressive strength with ten input parameters and did not include any other factors. However, a large database with an increased number of experiments and more input parameters, such as specimen size, curing age, etc., must be developed in the future for the utilized models to provide more accurate results.

## Figures and Tables

**Table 1:** Statistical checks of XGBoost, gradient-boosting, and random forest models.

Techniques	MAE (MPa)	RMSE (MPa)	R^2^
XGBoost	4.6	6.5	0.90
Gradient boosting	2.4	3.5	0.95
Random forest	2.4	3.1	0.96

## Data Availability

The data used in this research are properly cited and reported in the main text.
